# TRP120-dependent activation of noncanonical Wnt/NFAT signaling drives monocyte chemokine production in *Ehrlichia chaffeensis* infection

**DOI:** 10.1128/msphere.00081-26

**Published:** 2026-06-22

**Authors:** Regina N. Solomon, Duc-Cuong Bui, Ayana P. Pai, Jere W. McBride

**Affiliations:** 1 Department of Pathology, University of Texas Medical Branch 198642, Galveston, Texas, USA; 2 Department of Microbiology and Immunology, University of Texas Medical Branch 547647, Galveston, Texas, USA; 3 Center for Biodefense and Emerging Infectious Diseases, University of Texas Medical Branch 12338, Galveston, Texas, USA; 4 Sealy Institute for Vaccine Sciences, University of Texas Medical Branch 559814, Galveston, Texas, USA; 5 Institute for Human Infections and Immunity, University of Texas Medical Branch 551582, Galveston, Texas, USA; University at Buffalo-Downtown Campus, Buffalo, New York, USA

**Keywords:** *Ehrlichia*, NFAT transcription factors, short linear motif, tandem repeat protein, chemokines, infectious disease, cell signaling, monocyte recruitment

## Abstract

*Ehrlichia chaffeensis* is an obligately intracellular bacterium that repurposes multiple cellular signaling pathways, including Wnt, Hippo, Notch, and Hedgehog to evade innate immune defenses of mononuclear phagocytes. Although noncanonical Wnt signaling activation by *E. chaffeensis* induces nuclear factor of activated T-cells (NFAT) activity, the functional consequences of this response have remained unclear. Here, we demonstrate that *E. chaffeensis* exploits noncanonical Wnt/Ca^2+^ signaling to selectively activate NFATc1 and drive monocyte chemotactic chemokine production. In *E. chaffeensis-*infected THP-1 cells, NFATc1 was exclusively activated, which was recapitulated in cells treated with soluble recombinant TRP120 (rTRP120) or TRP120 Wnt ligand short linear motif (SLiM) peptide (QDVASH). Correspondingly, significant increases and similar chemokine expression profiles were detected in THP-1 cells infected with *E. chaffeensis* or treated with soluble rTRP120. Moreover, treatment with the NFATc1 peptide inhibitor (11R-VIVIT) significantly inhibited NFAT activation and chemokine expression. Consistent with a requirement for Wnt signaling, Frizzled 5 (FZD5)-deficient THP-1 cells exhibited reduced NFATc1 activation and diminished chemokine production and monocyte recruitment. Although the TRP120 Wnt ligand SLiM phenocopied *E. chaffeensis-*induced NFATc1 activation, it was insufficient to independently stimulate chemokine production. In contrast, ectopic expression of TRP120-induced chemokine expression, which was further amplified by TRP120 Wnt SLiM treatment, indicating both TRP120-directed extracellular Wnt signaling and intracellular mechanisms are required. Collectively, these findings define a previously unrecognized pathogen-driven strategy that co-opts non-canonical Wnt/NFAT signaling to promote monocyte chemotactic chemokine production.

**IMPORTANCE:**

*Ehrlichia chaffeensis* is an obligately intracellular bacterium that causes human monocytic ehrlichiosis and survives within mononuclear phagocytes by manipulating key cell signaling pathways. Unlike most Gram-negative bacteria, *E. chaffeensis* lacks classical pathogen-associated molecular patterns such as lipopolysaccharide and peptidoglycan, yet it stimulates strong chemokine production during infection; however, the molecular patterns, receptors and signaling pathways involved in inducing chemokine expression are unknown. Here, we uncover a cell signaling strategy whereby *E. chaffeensis*, through TRP120 Wnt ligand mimicry, co-opts the noncanonical Wnt/Ca^2+^ signaling to selectively activate NFATc1 and drive chemokine expression. This pathogen-driven NFAT activation promotes monocyte recruitment, revealing a previously unrecognized chemokine induction mechanism.

## INTRODUCTION

*Ehrlichia chaffeensis* is an obligately intracellular, Gram-negative bacterium that causes the life-threatening tick-borne zoonosis, human monocytic ehrlichiosis (HME) (1). *E. chaffeensis* infects mononuclear phagocytes where it replicates within cytoplasmic, membrane-bound vacuoles forming microcolonies known as morulae. To establish intracellular survival and persistence, *E. chaffeensis* has evolved sophisticated strategies to subvert innate immune defenses of mononuclear phagocytes, including co-opting host cell signaling pathways (2–6). Central to these host–pathogen interactions are tandem repeat protein (TRP) effectors, which are secreted via the type I secretion system and play critical roles in promoting monocyte infection (7–10).

TRP120 is a well-characterized multifunctional effector that functions extracellularly as a ligand mimic that engages multiple cell signaling receptors and intracellularly, primarily in the nucleus, where it functions as a transcription factor and ubiquitin ligase. Through these distinct yet complementary activities, TRP120 co-opts multiple cell signaling pathways, manipulates host transcription, and regulates key cellular networks, making it a central determinant of infection and pathogen survival (2–5). As a molecular mimic of multiple signaling pathway ligands, TRP120 contains short linear motifs (SLiMs) within its tandem repeat (TR) domain that activate evolutionarily conserved signaling pathways, including Wnt, Notch, Hedgehog, and Hippo. SLiMs are short (3–10 amino acids), linear interaction motifs that are typically located within intrinsically disordered protein domains (6). Interestingly, computational analysis using the Eukaryotic Linear Motif (ELM) resource identified at least 45 unique SLiM classes containing 189 individual motifs within TRP120 (7), highlighting its remarkable potential to interface with a broad spectrum of host cell targets.

We previously determined that TRP120 activates both canonical and noncanonical Wnt pathways to promote ehrlichial entry, inhibit autolysosome formation, and delay apoptosis through Wnt-Hippo crosstalk (8–10). Wnt signaling is an evolutionarily conserved pathway that regulates cell polarity, migration, development, and cell fate. In contrast to canonical Wnt signaling, the noncanonical pathways—Wnt/planar cell polarity (PCP) and Wnt/calcium (Ca^2+^)—remain poorly defined in the context of *E. chaffeensis* infection. The Wnt/PCP pathway regulates cell motility and polarity via Rho family GTPases (Rac, CDC42, and RhoA), which activate Jun-N-terminal kinase (JNK), AP-1, and Rho-associated kinase (ROCK) signaling (11). The Wnt/Ca^2+^ pathway activates phospholipase Cg (PLCg) resulting in phosphatidylinositol 4,5-biphosphate (PIP2) hydrolysis into inositol 1,4,5-triphosphate (IP3) and diacylglycerol (DAG), promoting increased levels of intracellular Ca^2+^ and subsequent activation of Ca^2+^-dependent proteins including protein kinase C (PKC), Ca^2+^/calmodulin-dependent kinase II (CaMKII), calcineurin (CaN), and nuclear factor of activated T-cells (NFAT) (11).

The NFAT family of transcription factors, evolutionarily related to REL/NF-kB family, consists of five members: NFATc1 (NFAT2/NFATc), NFATc2 (NFAT1/NFATp), NFATc3 (NFAT4/NFATx), NFATc4 (NFAT3), and NFAT5 (TonE-BP/OREBP). NFATs were initially identified in T cells but are now recognized to have functionality in both immune and non-immune cells (12). NFAT5 is a distantly related member primarily activated by osmotic stress, whereas NFATc1-4 are Ca^2+^/CaN-regulated and are maintained in the cytoplasm in a hyperphosphorylated state when inactive (13). Elevated intracellular Ca^2+^ activates CaN, which dephosphorylates NFATs, exposing their nuclear localization sequence (NLS) to facilitate nuclear entry. Within the nucleus, NFATs partner with transcriptional regulators such as AP-1, CBP/p300, and ICSBP/IRF8 to drive target gene expression (14–17).

NFAT signaling and transcription promote robust cytokine and chemokine induction. Lymphocytes lacking NFAT activity shows defective IL-2, IL-4, and CD40 expression (18). Moreover, NFAT-CaN inhibition reduces TNF-a, IL-10, and MCP-1 chemokine levels in primary human monocytes (PHMs) stimulated with fungal ligands or calcium ionophores (19). Notably, our previous work demonstrates that TRP120 binds a G + C rich consensus sequence and targets promoters of inflammatory chemotactic genes, linking its activity to infection-induced immunopathology (2). Additionally, we previously reported that *E. chaffeensis* activates noncanonical Wnt signaling and promotes NFATc1 activity, but the mechanism and pathogen-beneficial outcomes of NFAT activation are relatively undefined (4, 8).

In this study, we identify a previously unrecognized pathogen-driven mechanism of chemokine induction in which *E. chaffeensis* co-opts the noncanonical Wnt/Ca^2+^ signaling pathway through TRP120 Wnt SLiM ligand mimicry to selectively activate NFATc1 and stimulate chemokine production. We demonstrate that *E. chaffeensis*-mediated chemokine induction involves extracellular TRP120-dependent activation of the Wnt pathway, which synergizes with TRP120-directed NFAT transcriptional activity to drive chemokine gene expression and establish a chemokine profile that promotes monocyte migration. Defining this pathogen-directed signaling strategy provides new insight into how intracellular bacteria exploit host signaling and transcriptional programs to promote infection.

## RESULTS

### *E. chaffeensis* selectively activates NFATc1

**Fig 1:**
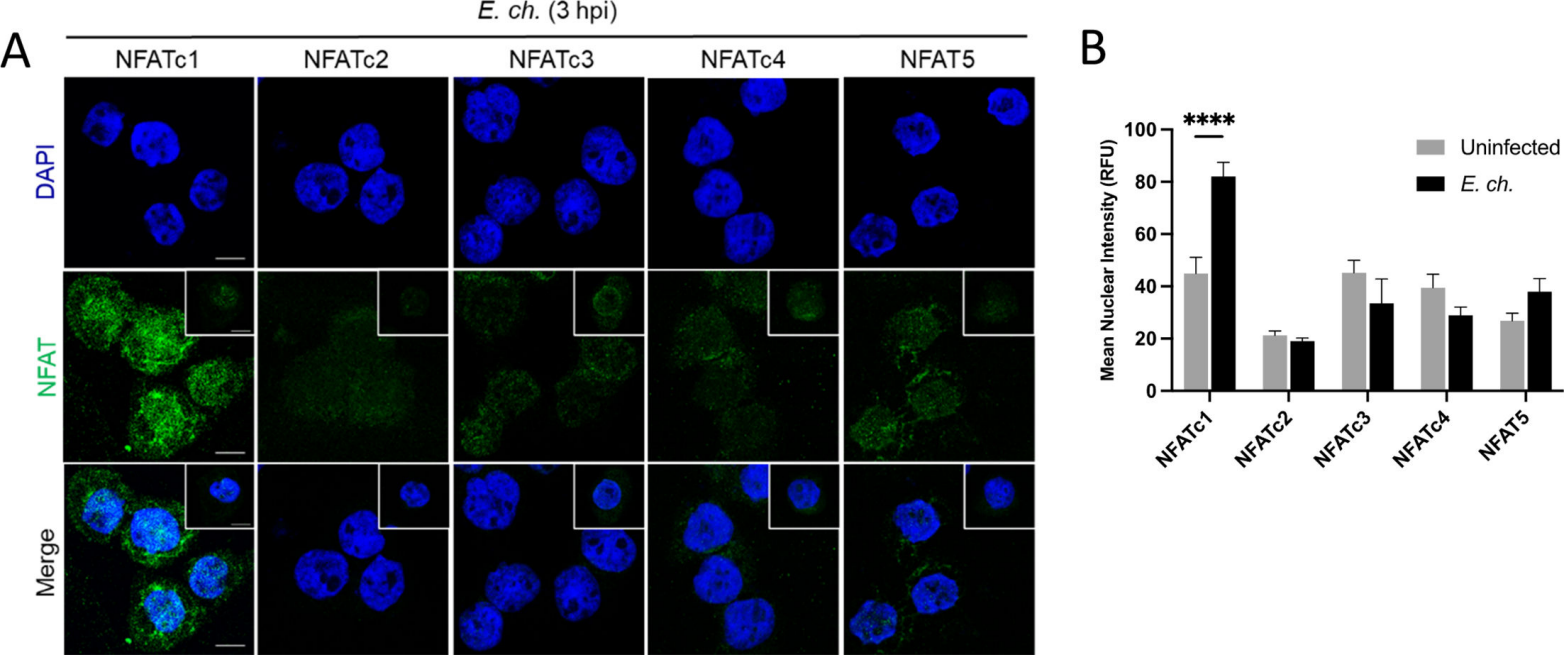
*E. chaffeensis* selectively activates NFATc1 during infection. (**A**) Immunofluorescent confocal microscopy of NFAT family member nuclear localization (green) in THP-1 cells after *E. chaffeensis* infection (3 h). Nucleus is stained with 4′,6-diamidino-2-phenylindole (DAPI; blue). Insets indicate uninfected control cells harvested prior to infection (Scale bar = 10 µm). (**B**) Nuclear intensity graph of NFAT average relative fluorescent units (RFU) compared to respective controls. ImageJ was used to measure mean pixel intensity of each cell across independent experiments based on regions of interest (ROI) defined by DAPI (*n* = >50). Statistical significance was calculated using two-tailed Student’s *t*-test, comparing each treatment condition to their respective control group. Data represent three experimental replicates and is presented as mean ± SD (****P < 0.0001).

We have previously reported NFATc1 activation and nuclear localization during *E. chaffeensis* infection (8), but the status of other NFAT family members has not been investigated. Thus, we examined activation and nuclear localization of all NFAT family members during *E. chaffeensis* infection. Confocal immunomicroscopic analysis revealed significant increases in NFATc1 activation and nuclear localization, while no significant changes in NFATc2, c3, c4, and NFAT5 activation were observed compared to uninfected control cells (Fig. 1A and B).

### *E. chaffeensis* rapidly activates NFATc1

**Fig 2:**
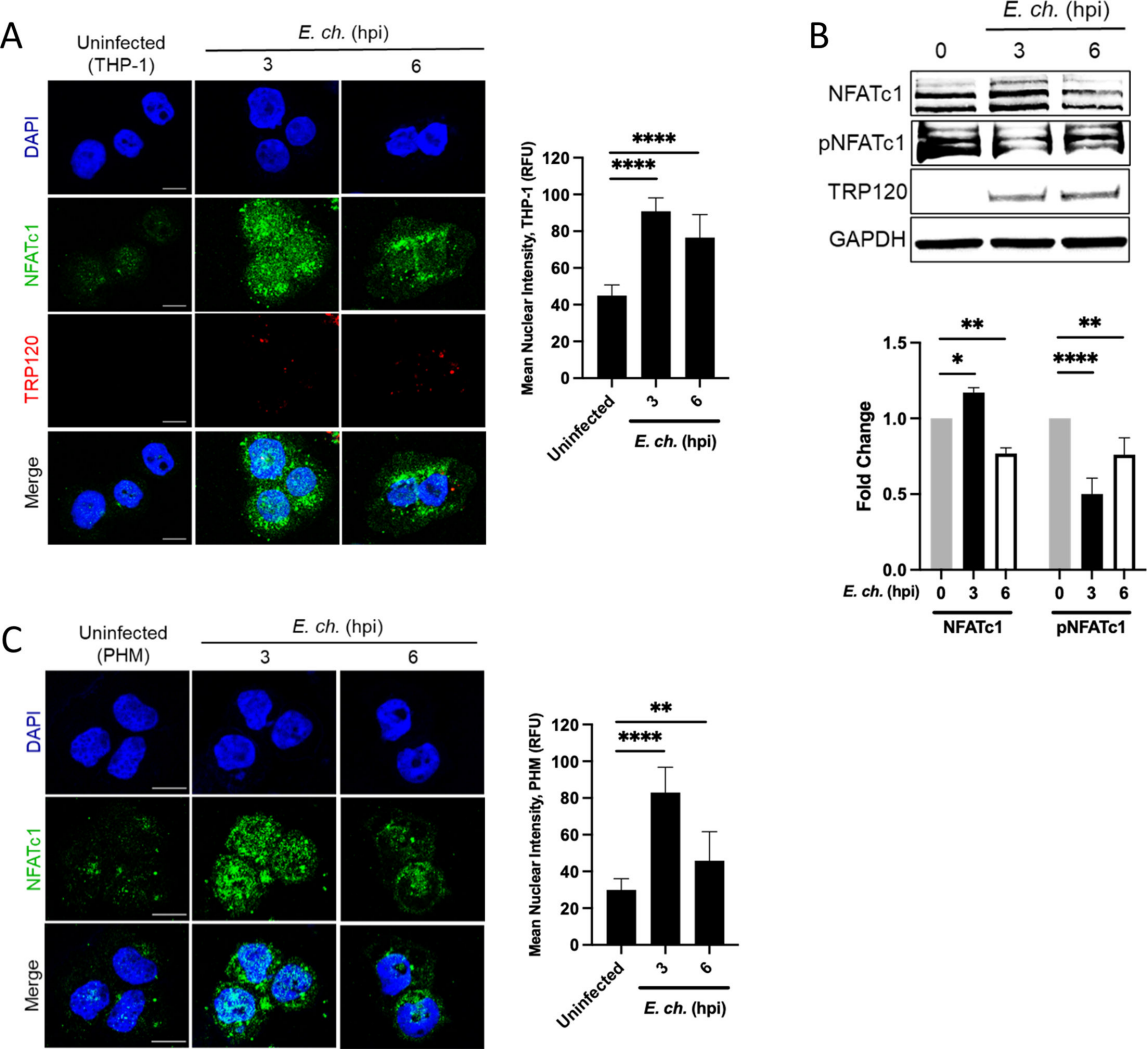
*E. chaffeensis* promotes NFATc1 nuclear localization in THP-1 cells and primary human monocytes. (**A**) Immunofluorescent confocal microscopy of *E. chaffeensis*-infected (MOI 100) THP-1 cells harvested at 0, 3, and 6 hpi and probed for NFATc1 (green) to examine nuclear accumulation and TRP120 (red) to confirm infection. (**B**) Western immunoblot of whole cell lysates and fold change analysis (bottom) of NFATc1 and pNFATc1 (inactive) normalized to GAPDH. (**C**) Immunofluorescent confocal microscopy demonstrating nuclear localization of NFATc1 in *E. chaffeensis*-infected (MOI 100) primary human monocytes (PHMs) harvested at 0, 3, and 6 hpi. (**A and C**) Mean fluorescent intensity graphs of NFATc1 nuclear accumulation compared to uninfected control. Analysis was performed using ImageJ to determine the mean pixel intensity of each cell across independent experiments (*n* = >50). Statistical significance was calculated using one-way ANOVA with Dunnett’s *post hoc* test, comparing each treatment condition to uninfected control. Data represent three experimental replicates and are presented as mean ± SD (*P < 0.05; **P < 0.01; ****P < 0.0001). Scale bar = 10 mm.

NFATc1 is a Ca2+-dependent transcription factor that localizes to the nucleus after dephosphorylation by calcineurin (CaN). *E. chaffeensis* is known to promote rapid induction of calcium shortly after infection (20); however, the downstream functional impact is not well understood. NFATc1 activation and nuclear localization increased significantly in *E. chaffeensis-*infected THP-1 cells and PHMs by 3 hpi compared to uninfected control cells (Fig. 2A and C). The 2–6 h interval for NFAT activation analysis after *E. chaffeensis* infection or TRP120 or Wnt SLiM treatment was selected based on a time-course study that delineated the period of peak NFAT activation during infection (Fig. S1). Increased NFATc1 levels were further confirmed by Western immunoblot analysis (Fig. 2B). Additionally, levels of pNFATc1 (inactive form) were also determined. There was a significant decrease in pNFATc1 expression at 3 h compared to uninfected cells and a detectable increase of pNFATc1 at 6 hpi (Fig. 2B).

### TRP120 and Wnt SLiM mimic phenocopy *E. chaffeensis*-mediated NFATc1 activation

**Fig 3:**
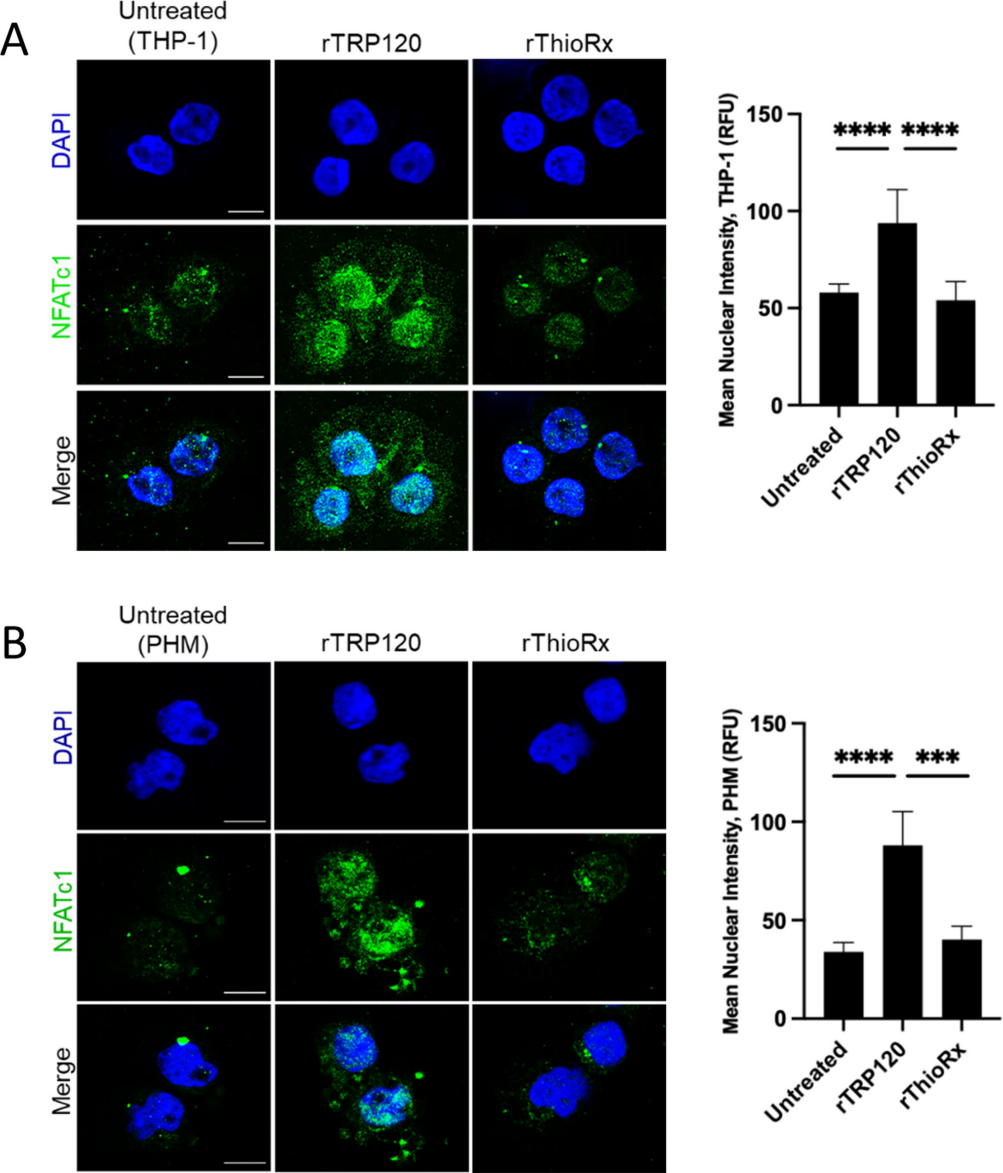
TRP120 phenocopies *E. chaffeensis*-induced NFATc1 activation. Immunofluorescent confocal microscopy of rTRP120 or rThioredoxin (rThioRx; negative control) treated (1 mg/mL) (**A**) THP-1 cells or (**B**) PHMs harvested 2 h post treatment (hpt) and stained with NFATc1 (Scale bar = 10 mm). (**A and B**) Mean fluorescent intensity graphs showing NFATc1 nuclear localization compared to uninfected control. Analysis was performed using ImageJ to determine the mean pixel intensity of each cell across three independent experiments (*n* = >50). Statistical significance was calculated using one-way ANOVA with Bonferroni’s *post hoc* test for multiple comparisons across treatment groups. Data represent three experimental replicates and presented as mean ± SD (***P < 0.001; ****P < 0.0001).

To examine TRP120-mediated NFATc1 activation, THP-1 cells and PHMs were treated with rTRP120 and analyzed via immunofluorescent confocal microscopy. NFATc1 nuclear localization was significantly higher 2 h post treatment (hpt) with TRP120 when compared to mean nuclear intensity of the thioredoxin (rThioRx) control (Fig. 3A and B). Collectively, these data demonstrate NFATc1 is rapidly activated after *E. chaffeensis* infection and TRP120 phenocopies *E. chaffeensis-*induced NFATc1 activation.

**Fig 4:**
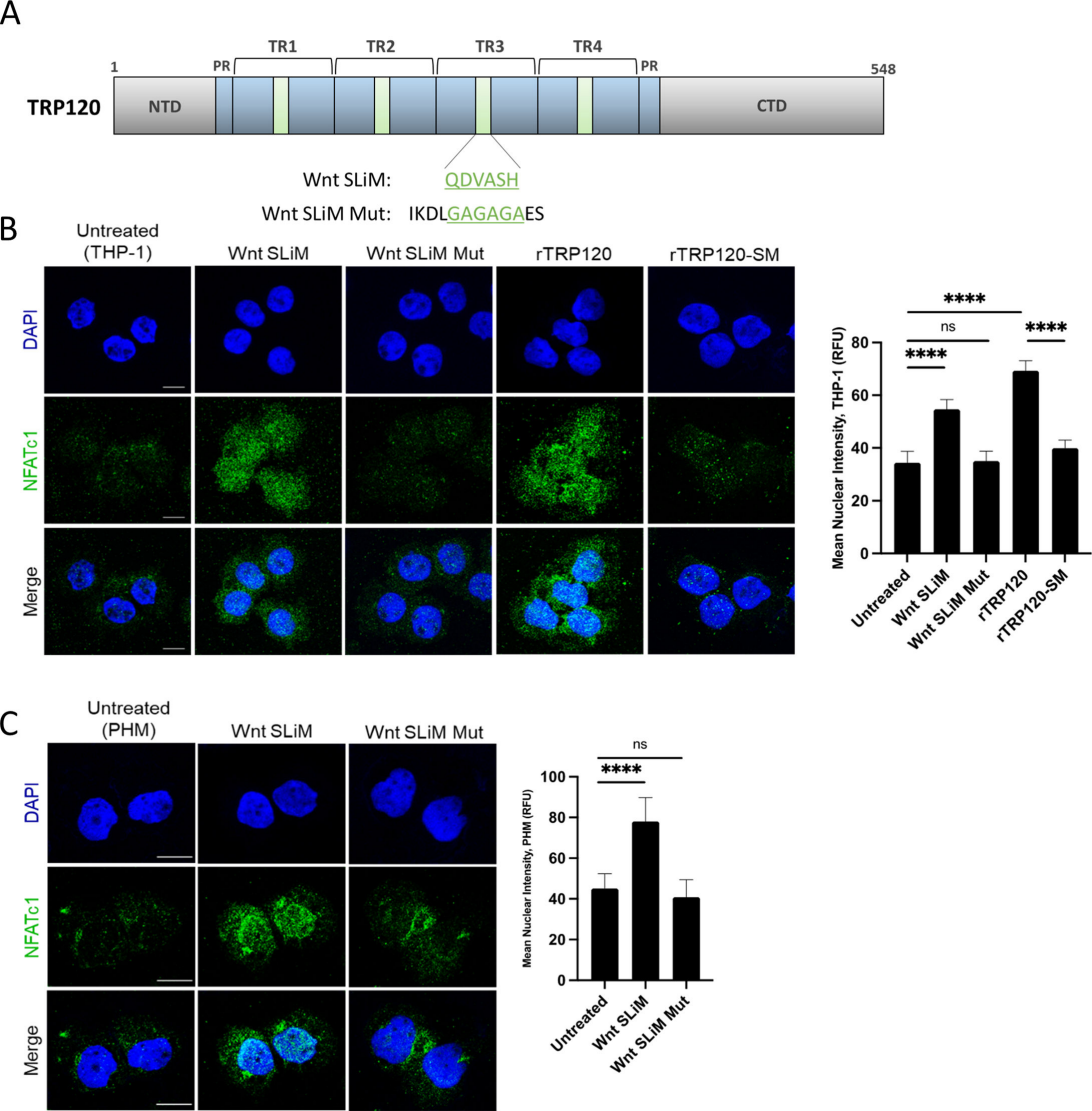
*E. chaffeensis* TRP120 Wnt SLiM ligand mimic phenocopies NFATc1 activation. (**A**) Schematic of TRP120 depicting location of the Wnt SLiM within each tandem repeat and the sequences used for Wnt SLiM and rTRP120 substitution mutant (rTRP120-SM). TR = Tandem repeat; PR = Partial repeat. (**B**) Immunofluorescent confocal microscopy of THP-1 cells probed for endogenous NFATc1 upon treatment with Wnt SLiM, Wnt SLiM mutant, rTRP120, or rTRP120-SM (1 mg/mL). (**C**) PHMs visualized by immunofluorescent confocal microscopy after Wnt SLiM and Wnt SLiM mutant treatment and probed for NFATc1 to detect nuclear localization. Wnt SLiM-treated cells were harvested 1 hpt, and recombinant protein-treated cells were harvested 2 hpt. (**B and C**) Mean fluorescent intensity graphs showing NFATc1 nuclear intensity compared to uninfected controls. Analysis was performed using ImageJ to determine the mean pixel intensity of each cell across independent experiments (*n* = >50). Statistical significance was calculated using one-way ANOVA followed by Bonferroni’s *post hoc* test for multiple comparisons or with Dunnett’s *post hoc* test, comparing each treatment condition to the uninfected control. Data represent three experimental replicates and presented as mean ± SD (ns = not significant, *P* > 0.05; ****P < 0.0001). Scale bar = 10 mm.

The TRP120 Wnt SLiM mimic is a well-characterized motif known to engage the Wnt and Hippo pathways during infection (4, 10). The SLiM is present within each repeat of the TR domain. The Wnt SLiM sequences within TRP120 and the motif used to generate the Wnt SLiM mutant peptide and rTRP120-substitution mutant (rTRP120-SM) are identified in the schematic (Fig. 4A). Expression and purification of mutant recombinant protein were verified by SDS-PAGE (Fig. S2). To investigate the role of the TRP120 Wnt SLiM in NFATc1 activation, THP-1 cells (Fig. 4B) and PHMs (Fig. 4C) were incubated with soluble Wnt SLiM peptide and examined by immunofluorescent confocal microscopy. In both cell types, NFATc1 nuclear accumulation increased significantly 1 hpt with the Wnt SLiM peptide compared to the Wnt SLiM mutant. To further define the involvement of TRP120 Wnt SLiM in NFATc1 activation during infection, a TRP120 mutant was generated in which the four Wnt SLiM repeats were replaced with glycine/alanine residues (Fig. 4A). In comparison to wild-type rTRP120-treated THP-1 cells, rTRP120-SM did not stimulate NFATc1 nuclear localization in THP-1 cells 2 hpt (Fig. 4B). Collectively, these data demonstrate the role of TRP120 Wnt SLiM in NFATc1 activation.

### TRP120 Wnt SLiM antibody neutralizes *E. chaffeensis*-mediated NFATc1 activation

**Fig 5:**
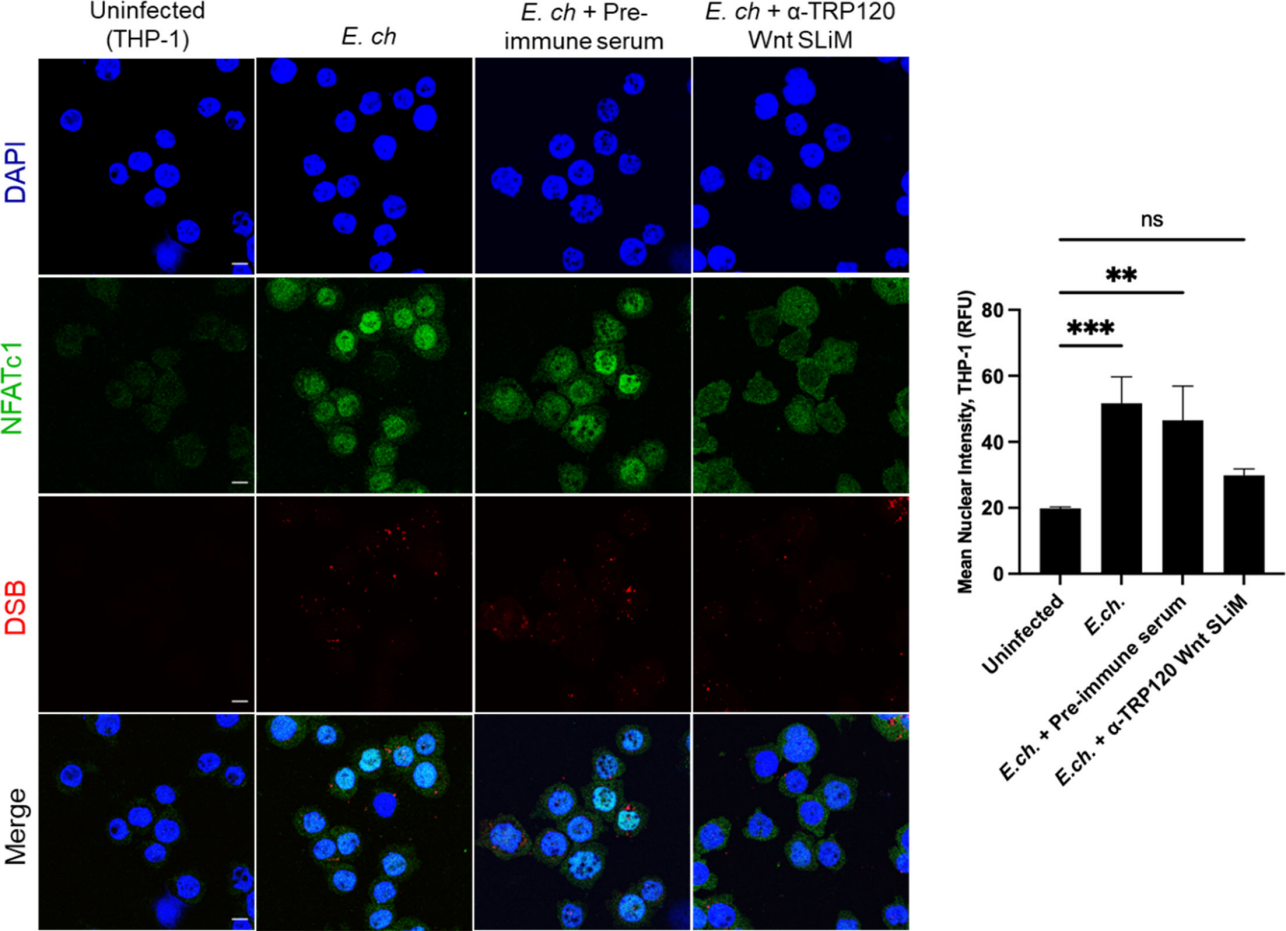
A TRP120 antibody specifically targets the Wnt SLiM motif and neutralizes NFATc1 activation during infection. Immunofluorescent confocal microscopy of *E. chaffeensis*-infected (MOI 100) THP-1 cells following a neutralization assay with an α-TRP120 Wnt SLiM antibody. All treatment groups were harvested 3 hpi and probed for NFATc1 nuclear localization. Mean fluorescent intensity graph depicts NFATc1 nuclear levels compared to the uninfected control group. *E. chaffeensis* was stained with anti-DSB to confirm infection. Analysis was performed using ImageJ to determine the mean pixel intensity of multiple cells per field of view (*n* = >50). Statistical significance was calculated using one-way ANOVA with Dunnett’s *post hoc* test, comparing each treatment condition to the uninfected control group. Data represent three experimental replicates and presented as mean ± SD (ns = not significant, *P* > 0.05; **P < 0.01;***P < 0.001). Scale bar = 10 mm.

To examine the contribution of the Wnt SLiM mimic on NFATc1 levels in the context of *E. chaffeensis* infection, we conducted a neutralization assay to determine if blocking the TRP120 Wnt SLiM with a targeted antibody would inhibit *E. chaffeensis*-mediated NFATc1 activation. THP-1 cells were infected with cell free *E. chaffeensis*, premixed with either rabbit TRP120-Wnt-SLiM antiserum or the negative control rabbit preimmune serum and incubated for 3 h. All treatment groups were harvested 3 hpi and visualized by immunofluorescent confocal microscopy. Compared to uninfected control cells, we detected a significant increase in NFATc1 nuclear localization in cells infected with *E. chaffeensis* alone or in addition to rabbit preimmune serum (Fig. 5). By contrast, THP-1 cells infected in combination with α-TRP120-Wnt-SLiM did not exhibit significant changes in NFATc1 nuclear localization compared to uninfected controls. These data support the conclusion that TRP120 Wnt SLiM mimic plays a central role in activating NFATc1 during *E. chaffeensis* infection.

### 11R-VIVIT is a potent inhibitor of NFATc1 activation during infection

**Fig 6:**
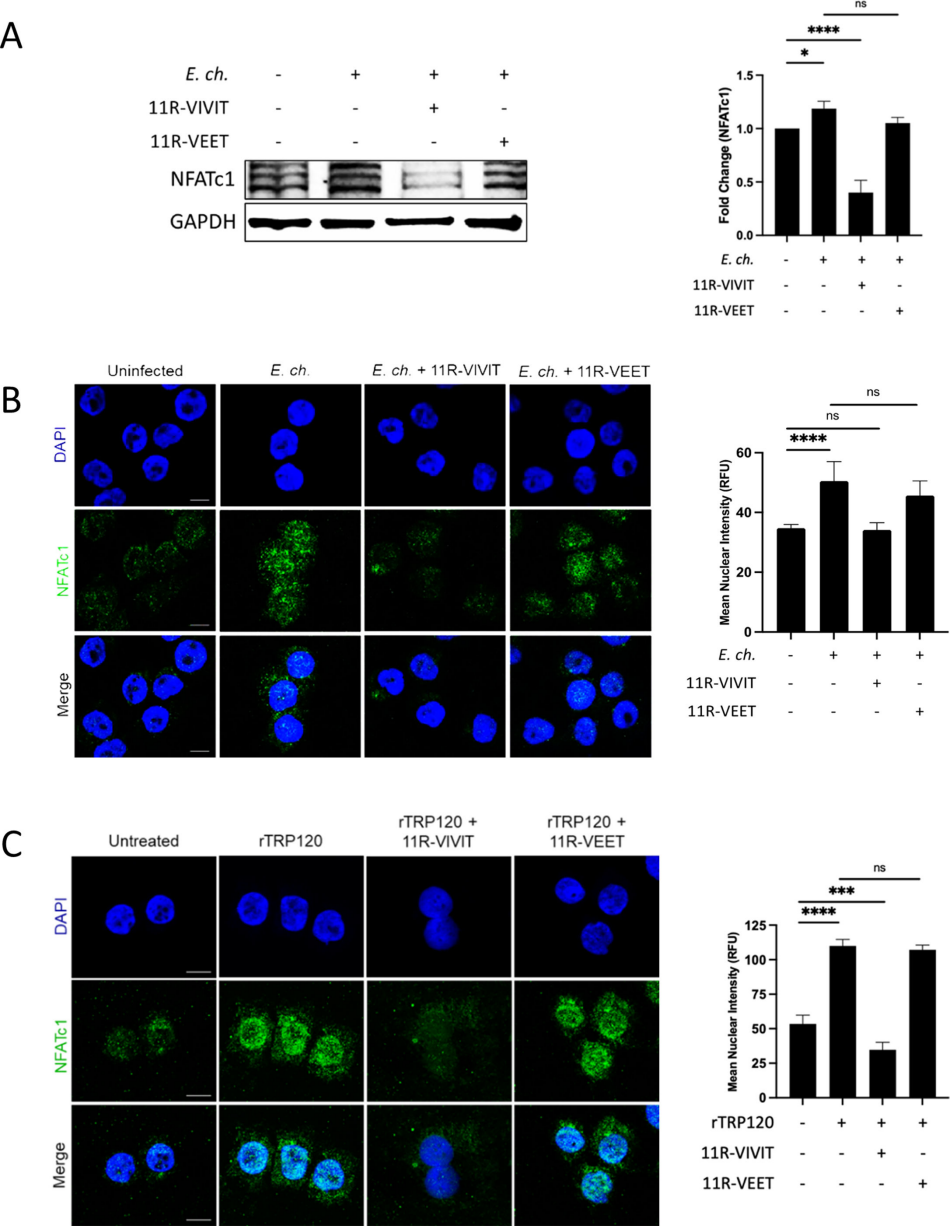
11R-VIVIT blocks *E. chaffeensis* TRP120-mediated NFATc1 activation. (**A**) Western immunoblot analysis of *E. chaffeensis*-infected THP-1 cells pre-treated with 11R-VIVIT inhibitor (1 h; 4 mM) or 11R-VEET, negative control peptide (1 h; 4 mM). (**B**) Immunofluorescent confocal microscopy of *E. chaffeensis*-infected THP-1 cells pre-treated with 11R-VIVIT or 11R-VEET negative control. (**C**) Immunofluorescent confocal microscopy of rTRP120-treated THP-1 cells with 11R-VIVIT or 11R-VEET pre-treatment. (**B and C**) Mean nuclear intensity graphs showing NFATc1 activation compared to uninfected controls. ImageJ analysis was used to determine the average pixel intensity of each cell across independent experiments (*n* = >50). Statistical significance was calculated using one-way ANOVA with Bonferroni’s *post hoc* test for multiple comparisons across treatment groups. Data represent three experimental replicates and presented as mean ± SD (ns = not significant, *P* > 0.05; *P < 0.05; **P < 0.01; ****P < 0.0001). Scale bar = 10 mm.

NFAT inhibitors are clinically administered as immunosuppressants; however, commonly used NFAT inhibitors such as cyclosporine A (CsA) and Tacrolimus (FK506) cause life-threatening side effects due to disruption of calcineurin (CaN) phosphatase activity (21–23). In this study, 11R-VIVIT, a recently established pharmacological inhibitor specific to NFATc1-4 (24–26), was used to antagonize *E. chaffeensis*-mediated NFATc1 activation. To characterize the effect of 11R-VIVIT on NFATc1 expression during infection, THP-1 cells were infected with *E. chaffeensis* following 1 h pre-treatment with 11R-VIVIT or 11R-VEET (negative control). Cell viability assays were conducted to determine the optimal inhibitor concentration for all experiments (Fig. S2). A significant decrease in NFATc1 expression was detected in cells treated with 11R-VIVIT compared to infected cells alone or *E. chaffeensis-*infected cells pre-treated with 11R-VEET (Fig. 6A). This observation was confirmed by immunofluorescent confocal microscopy showing basal levels (both nuclear and cytoplasmic) of NFATc1 in *E. chaffeensis*-infected cells pre-treated with 11R-VIVIT were similar to uninfected controls (Fig. 6B). To further define the efficacy of 11R-VIVIT during infection, THP-1 cells were treated with rTRP120 alone or after a 1 h pre-treatment with 11R-VIVIT or 11R-VEET. Consistent with previous results, NFATc1 nuclear localization significantly increased in cells treated with rTRP120 or 11R-VEET, but not in cells pre-treated with 11R-VIVIT (Fig. 6C). Collectively, these data establish 11R-VIVIT as an effective inhibitor for *E. chaffeensis* TRP120-mediated NFATc1 nuclear translocation.

### *E. chaffeensis* and rTRP120 upregulate chemokine gene expression

**Fig 7:**
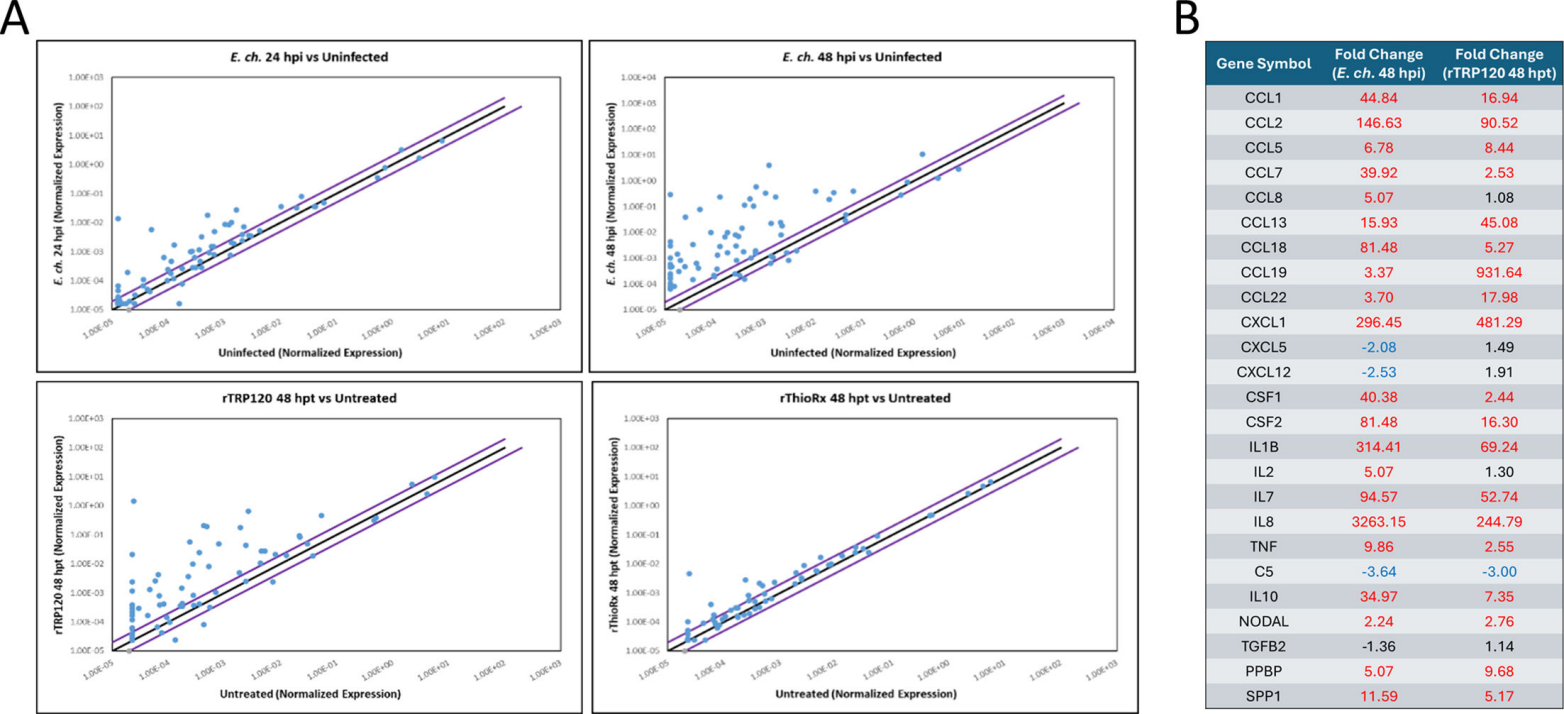
Chemokine gene expression in response to *E. chaffeensis* and TRP120. (**A**) Scatter plot graphs of 84 cytokines/chemokines expression analysis in THP-1 cells infected with *E. chaffeensis* (24 and 48 hpi; top) or treated with rTRP120 or rThioRx (48 h; bottom). Black lines indicate no change, and purple lines indicate twofold upregulation (above) or twofold downregulation (below) compared to uninfected controls. (**B**) Table of selected corresponding cytokine and chemokine genes with respective fold regulation of *E. chaffeensis*-infected cells (48 hpi). Red values = upregulated genes; Blue values = Downregulated genes; Black values = Unchanged genes. Representative data of at least three independent experiments are shown (*n* = 3).

We investigated chemokine expression profiles induced by *E. chaffeensis* and rTRP120 using a RT^2^ Profiler Human Cytokine & Chemokine PCR array (Table S1). All treatment groups were analyzed and plotted against their respective uninfected control group. Scatter plot data revealed increased gene expression levels in *E. chaffeensis*-infected THP-1 cells at 48 h compared to 24 h, indicating temporal increases over the course of infection (Fig. 7A). Based on peak gene transcription activity, we compared expression profiles between *E. chaffeensis* and rTRP120 at 48 h which revealed a significant increase in many CC family chemokines and interleukin (IL) cytokines, including *CCL2*, *CCL5*, *CCL7*, *CCL13*, *IL1b*, and *IL8* in both *E. chaffeensis-*infected and rTRP120-treated cells (Fig. 7B). THP-1 cells treated with rThioRx at 48 h were included to control for recombinant protein treatment.

### NFATc1 activation promotes chemokine induction and monocyte recruitment

**Fig 8:**
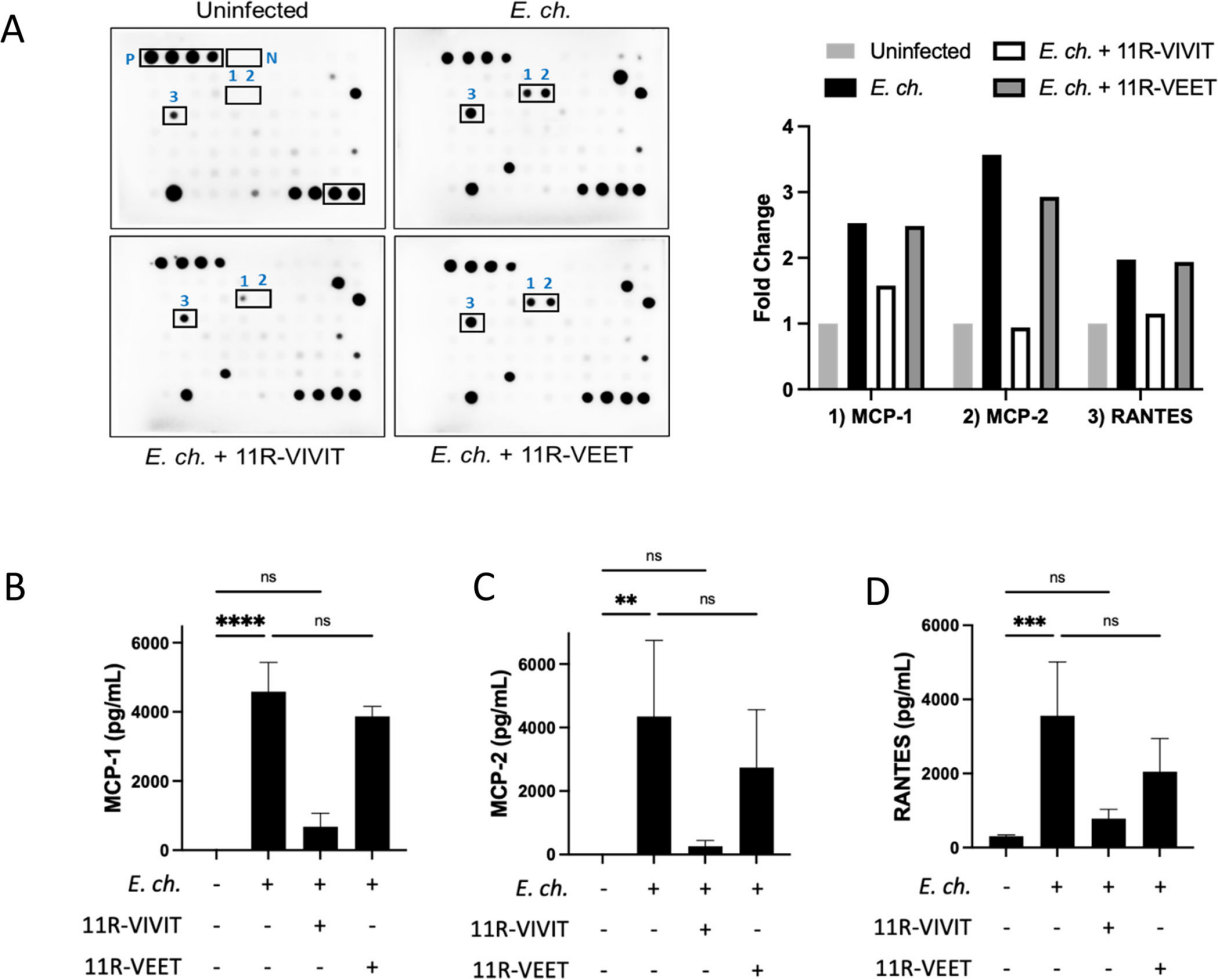
NFATc1 inhibition suppresses chemokine secretion during *E. chaffeensis* infection. (**A**) Dot blot membrane array incubated with conditioned media from *E. chaffeensis*-infected THP-1 cells pre-treated with 11R-VIVIT inhibitor (1 h; 4 mM) or 11R-VEET (1 h; 4 mM; negative control inhibitor). P = Positive control; N = Negative control. Graph represents fold change densitometry analysis of chemokine levels from each treatment group compared to their uninfected controls. (**B–D**) Enzyme linked immunosorbent assays (ELISA) of select chemokines from panel A. *E. chaffeensis*-infected THP-1 cells pre-treated with 11R-VIVIT or 11R-VEET measuring MCP-1, MCP-2, or RANTES. Statistical significance was calculated using one-way ANOVA with Bonferroni’s *post hoc* test for multiple comparisons across treatment groups. Data represent three experimental replicates and presented as means or mean ± SD (ns = not significant, *P* > 0.05; **P < 0.01; ***P < 0.001; ****P < 0.0001).

To examine the chemokines secreted by *E. chaffeensis-*infected THP-1 cells, conditioned media from uninfected and *E. chaffeensis*-infected THP-1 cells were harvested 48 hpi and screened using a membrane-based dot blot array printed with capture antibodies for 80 target chemokines/cytokines (Table S2). Compared to the uninfected control, cytokines and chemokines strongly secreted from *E. chaffeensis-*infected cells included CXCL1, IL-8, MCP-1, MCP-2, RANTES, and CXCL10. To examine which chemokines/cytokines were regulated by NFATc1, the NFATc1-specific inhibitor, 11R-VIVIT, was used to block NFAT activation. In response to 11R-VIVIT treatment, decreased levels of MCP-1, MCP-2, and RANTES were observed compared to *E. chaffeensis* infection alone or 11R-VEET (negative control) (Fig. 8A). Chemokines that exhibited decreased expression in response to 11R-VIVIT were further quantified via enzyme linked immunosorbent assays (ELISA). NFATc1 inhibition resulted in a significant reduction of MCP-1, MCP-2, and RANTES (Fig. 8B through D), confirming a primary role for NFATc1 in chemokine induction during infection.

**Fig 9:**
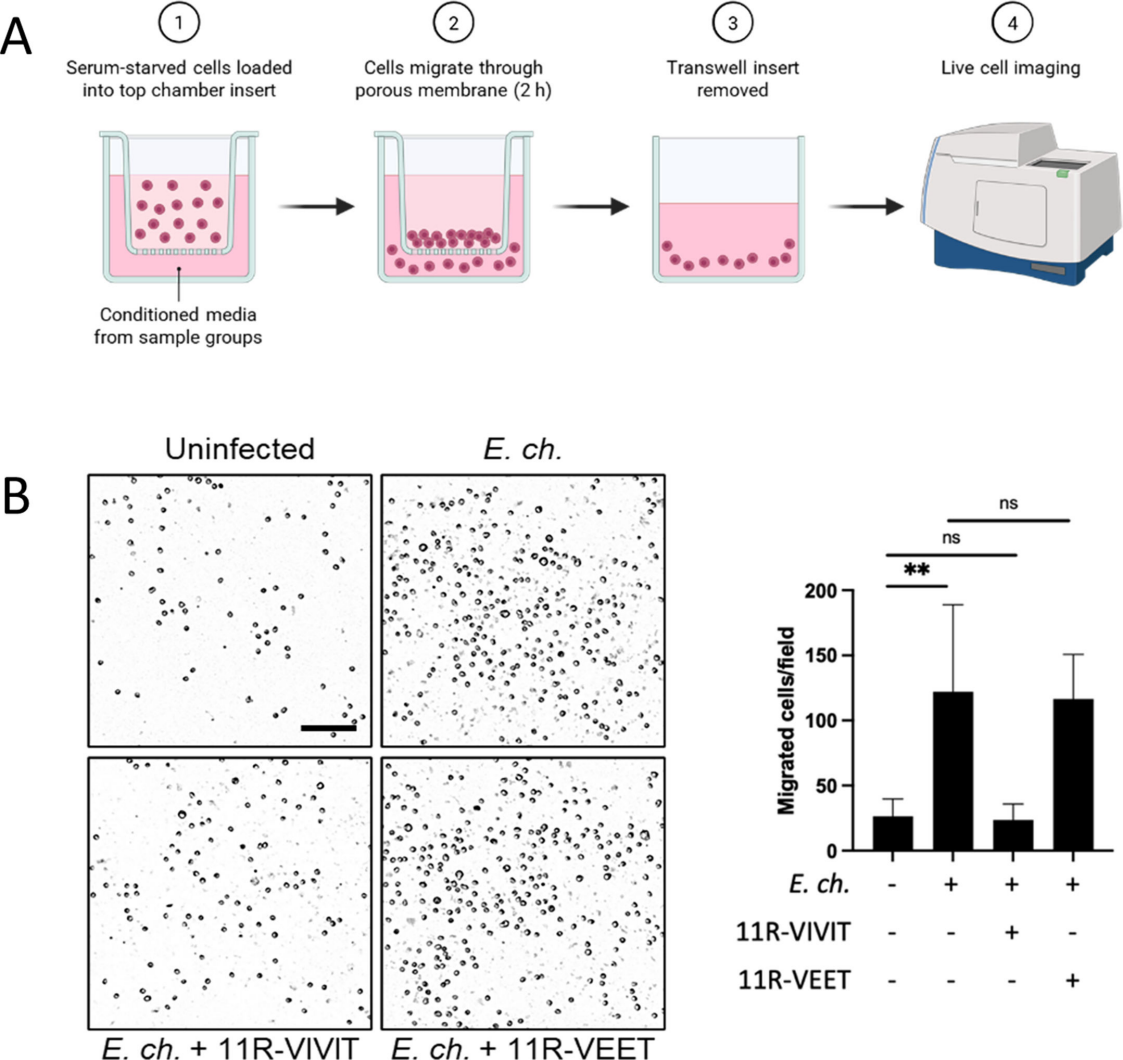
Monocyte recruitment is driven by *E. chaffeensis*-mediated NFATc1 activation. (**A**) Schematic of transwell migration assay protocol. Conditioned media from uninfected THP-1 cells, *E. chaffeensis*-infected cells only or *E. chaffeensis*-infected cells pre-treated with 11R-VIVIT inhibitor or 11R-VEET were seeded in bottom chambers. Serum-starved THP-1 cells were placed in top chamber inserts and incubated for 2 h. (**B**) Live cell imaging capturing the bottom chamber of transwell migration assay after a 2 h incubation (4× magnification). Representative fields are shown and graph depicts average number of migrated cells/field (*n* = 5). Statistical significance was calculated using one-way ANOVA with Bonferroni’s *post hoc* test for multiple comparisons. Data represent three experimental replicates and presented as mean ± SD (ns = not significant, *P* > 0.05; **P < 0.01). Scale bar = 50 mm.

Monocyte migration assays were used to model the impact of NFATc1-mediated chemokine induction on monocyte recruitment as depicted in the schematic of the transwell migration assay (Fig. 9A). After a 2 h incubation, live cell imaging was performed showing a significant decrease (81%) in cells that migrated toward conditioned medium harvested from infected cells pre-treated with 11R-VIVIT, compared to infection alone. There were no significant differences in cell migration between infection alone and infection pre-treated with 11R-VEET (Fig. 9B). These data suggest a pivotal role for NFATc1 activation during infection and reveal that *E. chaffeensis* exploits noncanonical Wnt signaling to initiate a pathogen-directed chemokine response.

### NFATc1 activation is dependent on the FZD5 receptor

**Fig 10:**
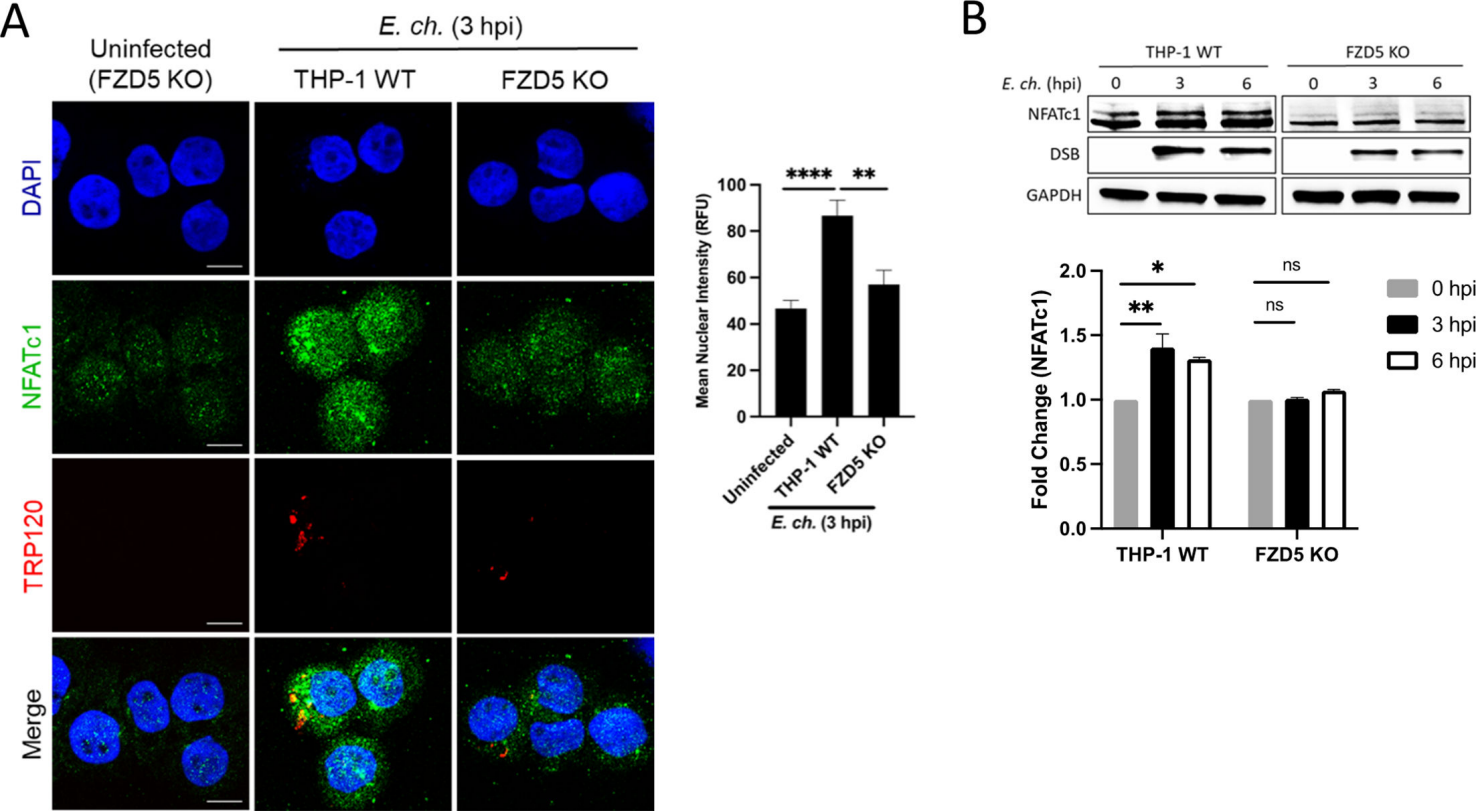
NFATc1 activation is mediated by FZD5. (**A**) Immunofluorescent confocal microscopy of uninfected FZD5 KO cells compared to *E. chaffeensis*-infected THP-1 cells or FZD5 KO cells (3 h) stained with NFATc1 (green) and TRP120 (red) to confirm infection (Scale bar = 10 mm). Mean nuclear intensity graph shows NFATc1 nuclear localization of each experiment group. ImageJ analysis tool was used to determine the average pixel intensity of cells across independent experiments (*n* = >50). Statistical significance was calculated using one-way ANOVA with Bonferroni’s *post hoc* test for multiple comparisons. Data collected from three experimental replicates and represented as means ± SD (ns = not significant, *P* > 0.05; **P < 0.01; ****P < 0.0001). (**B**) Western immunoblot and fold change analysis (bottom) of NFATc1 from whole cell lysates collected from *E. chaffeensis*-infected THP-1 cells or FZD5 KO cells harvested at 0, 3, and 6 hpi. *E. chaffeensis* was stained with anti-DSB to confirm infection. Statistical significance was calculated using one-way ANOVA with Dunnett’s *post hoc* test, comparing each treatment condition to their uninfected control group. Data represent at least three experimental replicates and presented as mean ± SD (ns = not significant, *P* > 0.05; *P < 0.05; **P < 0.01; ****P < 0.0001).

In previous studies, we have shown that the TRP120 Wnt SLiM engages FZD5 to stimulate downstream pathway activation (4, 8). To test the dependence on FZD5 for NFATc1 activity, FZD5 knockout (KO) cells that exhibited weak “leaky” FZD5 expression were used to examine its effect on NFATc1 activation (Fig. S4). Immunofluorescent confocal microscopy was used to visualize *E. chaffeensis*-infected wild-type THP-1 and FZD5 KO cells. Consistent with results from this study, NFATc1 demonstrated significant nuclear accumulation in THP-1 cells 3 hpi, but this response was significantly reduced in *E. chaffeensis*-FZD5 KO cells (Fig. 10A). These findings were confirmed by Western immunoblot analysis of whole cell lysates which found no significant changes in NFATc1 temporal expression in *E. chaffeensis*-infected FZD5 KO cells (Fig. 10B).

### Chemokine secretion and monocyte recruitment are diminished in FZD5 KO cells

**Fig 11:**
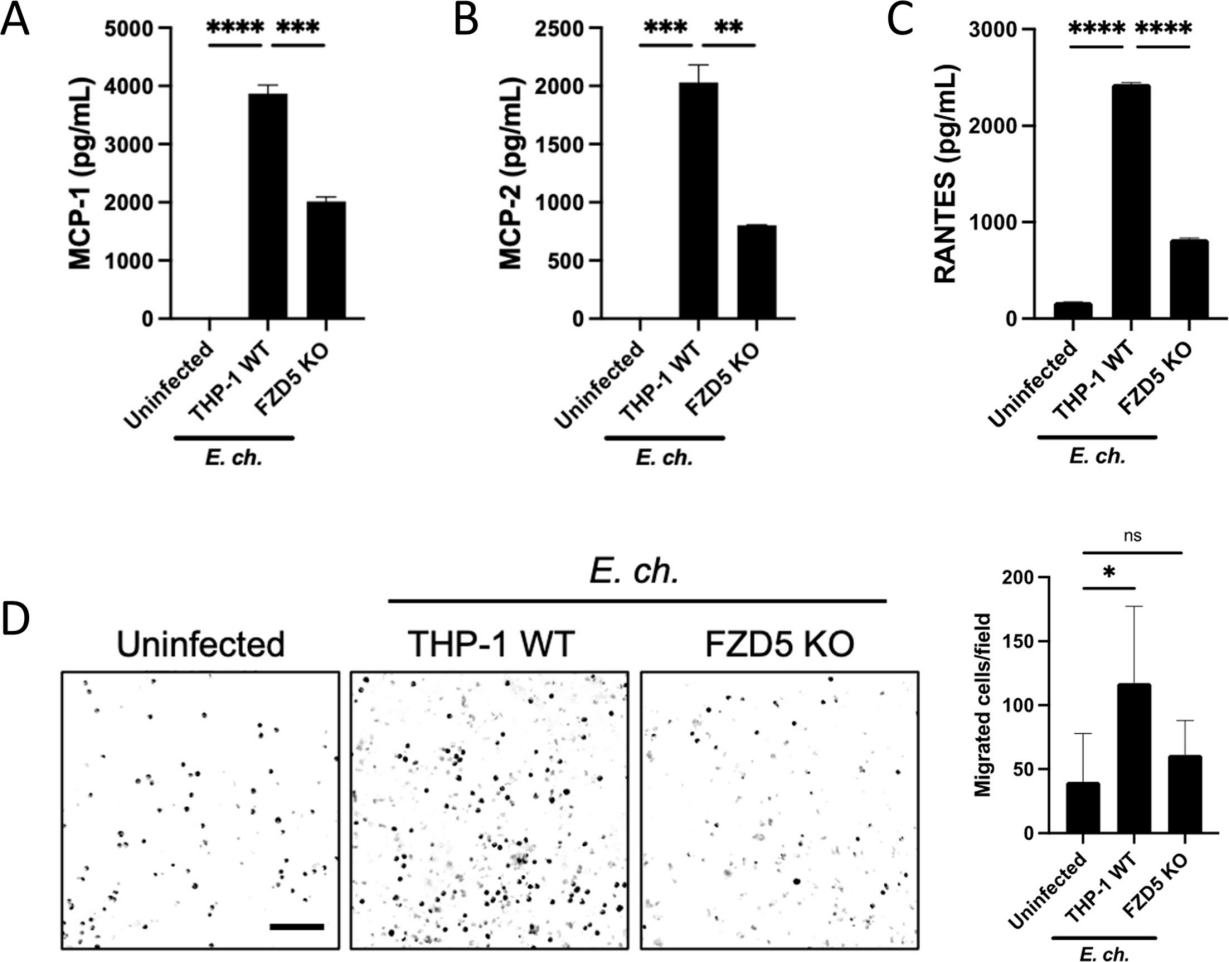
Chemokine secretion and monocyte migration is reduced in FZD5 KO cells during *E. chaffeensis* infection. ELISA to measure chemokine levels of (**A**) MCP-1, (**B**) MCP-2, and (**C**) RANTES from conditioned media harvested from uninfected THP-1 cells and *E. chaffeensis*-infected THP-1 cells or FZD5 KO cells harvested 48 hpi. Statistical significance was determined using one-way ANOVA with Bonferroni’s *post hoc* test for multiple comparisons. (**D**) Live cell imaging displaying bottom chamber of transwell migration assay from experimental groups as described above (4× magnification). Graph depicts average numeration of migrated cells/field (*n* = 5). Statistical significance was calculated using one-way ANOVA with Dunnett’s *post hoc* test, comparing each treatment condition to the uninfected control group. Data represent three experimental replicates and presented as mean ± SD (ns = not significant, *P* > 0.05; *P < 0.05, **P < 0.01, ***P < 0.001, ****P < 0.0001). Scale bar = 50 mm.

To determine the role of FZD5 receptor on *E. chaffeensis*-mediated chemokine secretion and monocyte migration, an ELISA was used to quantify chemokine concentrations in conditioned media harvested from *E. chaffeensis*-infected wild-type THP-1 and FZD5 KO cells. Significant reductions in MCP-1, MCP-2, and RANTES levels were detected from *E. chaffeensis-*infected FZD5 KO cells (Fig. 11A through C), compared to THP-1 WT cells. Moreover, a substantial but non-significant decrease (48%) in monocyte migration was observed in conditioned media harvested from *E. chaffeensis*-infected FZD5 KO cells 48 hpi (Fig. 11D), demonstrating that FZD5 plays a primary role in NFATc1 activation.

### Wnt SLiM requires ectopic expression of TRP120 to induce chemokine secretion

**Fig 12:**
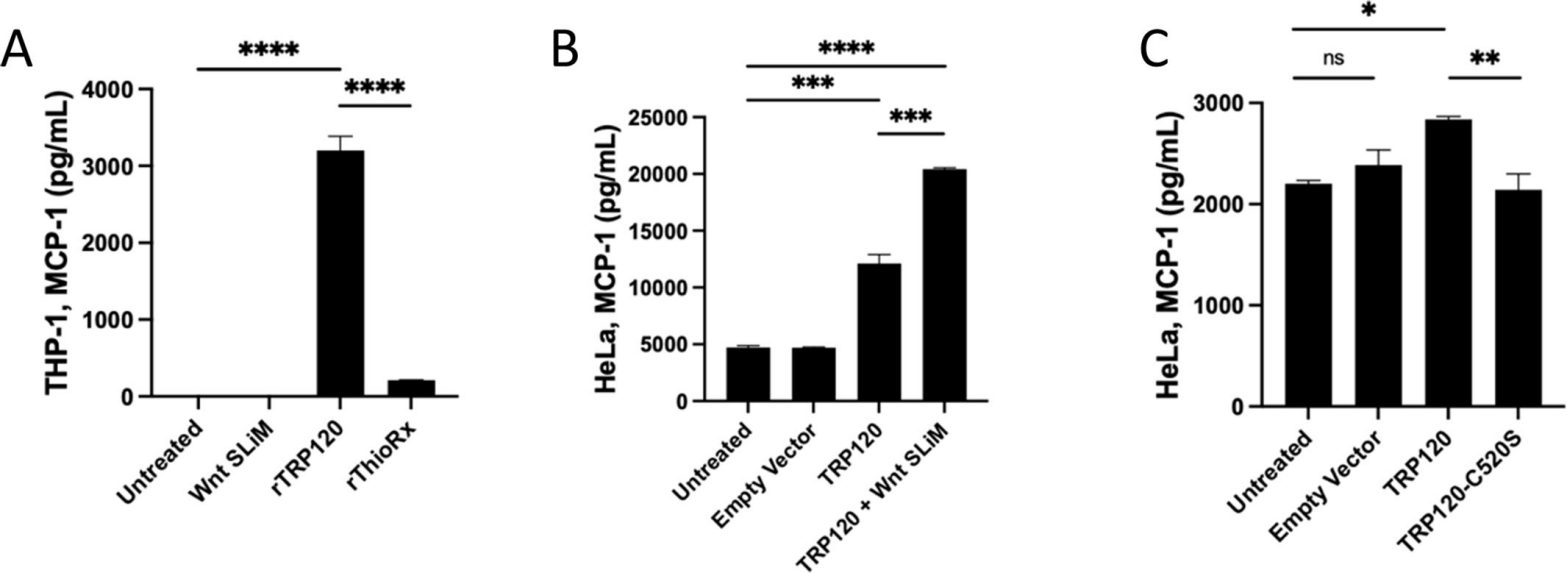
Wnt SLiM NFAT activation and intracellular TRP120 expression induce chemokine expression. MCP-1 ELISA measuring chemokine levels from conditioned media harvested from (**A**) untreated THP-1 cells or treated with Wnt SLiM (1 mg/mL), rTRP120 (1 mg/mL), or rThioRx (1 mg/mL, negative control). (**B**) HeLa cells transfected with GFP-Empty Vector, GFP-TRP120, or GFP-TRP120 stimulated with soluble Wnt SLiM mimic (1 mg/mL) to activate NFATc1. (**C**) HeLa cells transfected with 6xHis-Empty Vector, 6xHis-TRP120, or 6xHis-TRP120-C520S, catalytically inactive ubiquitin ligase mutant. Statistical significance was determined using one-way ANOVA with Bonferroni’s *post hoc* test for multiple comparisons across treatments groups. Data represent three experimental replicates and presented as mean ± SD (ns = not significant, *P* > 0.05; *P < 0.05, **P < 0.01, ***P < 0.001, ****P < 0.0001).

In this study, we have found that the TRP120 Wnt SLiM ligand mimic is critical to initiate NFATc1 signaling and that NFATc1 activation is a key signaling component upstream of chemokine production. To determine the sole contribution of the Wnt SLiM on chemokine induction, THP-1 cells were treated with the soluble Wnt SLiM ligand peptide (1 μg/mL), rTRP120 (1 μg/mL), or rThioRx-treated cells. Interestingly, conditioned media harvested 48 hpt revealed that MCP-1 levels did not increase in response to the TRP120 Wnt SLiM alone similar to the uninfected controls. However, rTRP120-treated THP-1 cells produced significant levels of MCP-1 compared to rThioRx-treated control cells (Fig. 12A). These results suggest that while the TRP120 Wnt SLiM is important for NFATc1 activation and nuclear translocation, there are additional TRP120-driven factors that contribute to NFATc1-mediated chemokine induction. To examine this question, HeLa cells were treated with soluble Wnt SLiM to activate NFATc1 in combination with TRP120 ectopic expression (Fig. 12B). Under these conditions, MCP-1 levels were significantly increased compared to TRP120 transfection alone, indicating that NFATc1 activation synergizes with TRP120 intracellular activity to promote chemokine expression.

Our laboratory has shown that TRP120 repurposes host machinery through distinct functional domains to promote infection (2, 9, 27–32). Notably, TRP120 encodes a C-terminal HECT E3 ubiquitin ligase domain that targets host regulators for degradation, including FBW7, a negative regulator of c-Jun/AP-1 (27). Because c-Jun cooperates with NFATc1 to drive cytokine and chemokine gene transcription, the absence of TRP120 ubiquitin ligase activity with Wnt SLiM treatment alone may permit FBW7 to remain active, limiting c-Jun stabilization and constraining NFATc1:AP-1 convergence. To examine the role of the TRP120 HECT E3 ubiquitin ligase in chemokine production, MCP-1 chemokine levels were measured in conditioned medium harvested from HeLa cells transfected with TRP120 alone or a catalytically inactive ubiquitin ligase mutant, TRP120-C520S. Our results demonstrate the level of MCP-1 induction was significantly reduced in the mutant transfected cells compared to wild-type TRP120 (Fig. 11C). Transfection efficiency of all TRP120 constructs were verified via western blot analysis (Fig. S5). Collectively, these findings identify the Wnt SLiM as a key signal for NFATc1 activation and indicate that robust chemokine production arises from a coordinated cascade integrating both extracellular and intracellular TRP120-mediated interactions.

## DISCUSSION

In previous investigations, we demonstrated that *E. chaffeensis* activates both canonical and noncanonical Wnt signaling pathways to promote intracellular infection (8). In the present study, we expand this framework by defining how *E. chaffeensis* co-opts host Wnt signaling to drive pathogen-beneficial chemokine responses. Central to this process is the multifunctional effector TRP120, which functions not only as an adhesin to promote phagocytosis and bacterial entry but also as a ligand mimic that activates Wnt signaling (4, 8). We previously identified TRP120 as the first bacterial Wnt ligand mimic, demonstrating that it engages the FZD5 receptor to activate both canonical and noncanonical pathways, including the Wnt/Ca^2+^ axis leading to NFAT nuclear localization (8). In the present study, we show that *E. chaffeensis* selectively activates NFATc1, and this response was phenocopied by recombinant TRP120 and the TRP120 Wnt SLiM peptide. Additionally, anti-TRP120 Wnt SLiM antibody blocked NFATc1 activation in response to *E. chaffeensis* infection. These findings strongly support a model in which TRP120 functions as a SLiM-based mimic that directly activates the noncanonical Wnt/Ca^2+^ signaling during infection.

A major finding of this study is that *E. chaffeensis* induces rapid NFATc1 nuclear translocation early during infection via activation of the noncanonical Wnt/Ca^2+^ pathway. While NFAT signaling is targeted by other intracellular pathogens including *Helicobacter pylori* (33, 34), the upstream mechanisms driving NFAT activation in ehrlichiosis have remained poorly defined. Here, we show that *E. chaffeensis* specifically induces NFATc1 nuclear localization in both THP-1 and PHMs, extending the conclusions from our previous observations (8). These findings are supported by prior reports demonstrating that *E. chaffeensis* infection rapidly induces calcium flux required for bacterial internalization and growth (20), thereby providing a mechanistic link between Wnt/Ca^2+^ signaling and NFAT activation. Importantly, we demonstrate that NFATc1 activation is mediated by the TRP120 Wnt SLiM. Both recombinant TRP120 and the soluble Wnt SLiM were sufficient to induce NFATc1 nuclear localization, whereas the deletion of the Wnt SLiM abolished this response, demonstrating the specificity of this motif. Given that TRP120 contains multiple SLiMs that engage diverse signaling pathways, these results highlight the Wnt SLiM as a critical functional motif driving NFAT activation. More broadly, these findings reinforce the concept that TRP120 acts as a modular signaling platform that orchestrates host cell reprogramming through discrete linear motifs.

To define the functional significance of NFATc1 activation, we utilized the selective NFAT inhibitor 11R-VIVIT. This peptide inhibitor mimics the conserved NFATc docking domain (PxIxIT) and competitively binds calcineurin to disrupt NFAT-calcineurin interactions without broadly impairing phosphatase activity (24–26). This approach avoids the off-target effects associated with classical inhibitors such as cyclosporin A and FK506. We included the scrambled control peptide, 11R-VEET, to further confirm functional specificity of 11R-VIVIT (35–38). Inhibition of NFATc1 with 11R-VIVIT effectively suppressed NFATc1 activation in *E. chaffeensis*-infected and rTRP120-treated THP-1 cells, validating its use as a specific tool for interrogating NFAT in this system. Interestingly, in response to 11R-VIVIT treatment, we consistently observed reductions in NFATc1 expression that fell below untreated control groups. This observation is likely related to this NFAT family member’s unique ability to increase its expression via feed-forward transcriptional auto-amplification (39), which appears to occur at baseline based on western immunoblot analysis. It is likely that *E. chaffeensis* infection triggers an increase in NFATc1-dependent *NFATc1* gene transcription and that baseline NFATc1 transcription is blunted at baseline levels in response to 11R-VIVIT (39, 40).

Functionally, NFATc1 inhibition significantly reduced secretion of key *E. chaffeensis* and TRP120 induced chemokines, including MCP-1, MCP-2, and RANTES, and impaired monocyte migration. In contrast, other chemokines, such as IL-8 and members of the Gro family, were unaffected, indicating selective regulation of chemokine subsets by NFATc1. These findings support a model in which *E. chaffeensis* selectively co-opts NFATc1-dependent transcriptional programs to drive selective chemokine responses that promote recruitment of permissive host cells while maintaining broader inflammatory signaling. This is consistent with clinical and experimental observations showing elevated levels of MCP-1, IL-8, RANTES, and other inflammatory mediators during HME (41), as well as a broad array of cytokine and chemokine induction in infected cells and animal models. (42, 43). However, prior studies have not identified a direct bacterial mechanism driving these responses. Our finding provides a mechanistic framework linking bacterial signaling to selective chemokine induction.

Our previous studies demonstrate that the TRP120 Wnt SLiM engages the FZD5 receptor to activate Wnt and Hippo signaling, thereby facilitating *E. chaffeensis* infection (4, 10). Notably, FZD5 is known to mediate inflammatory responses upon activation by Wnt5a, an endogenous Wnt ligand with sequence homology and functional similarity to the TRP120 Wnt SLiM (4, 44–46). Building on these observations, we investigated whether *E. chaffeensis*-induced NFATc1 activation and monocyte recruitment depend on FZD5. Indeed, NFATc1 nuclear localization was significantly reduced in FZD5 KO cells, and FZD5 silencing significantly diminished MCP-1, MCP-2, and RANTES secretion. Consistent with these findings, conditioned medium from *E. chaffeensis*-infected FZD5 KO cells exhibited reduced capacity to recruit monocytes.

Interestingly, while the TRP120 Wnt SLiM was sufficient to induce NFATc1 nuclear localization, it did not independently drive chemokine production. This indicates that NFATc1 activation alone is insufficient for full transcriptional induction and suggests that additional bacterial factors or host co-regulators are required. One possibility is cooperative regulation at enhancer elements. We previously reported that TRP120 functions as nucleomodulin and genome-wide mapping of TRP120-DNA interactions identified extensive binding across the host genome, including regulatory regions of chemokine genes (2), raising the possibility that TRP120 and NFATc1 converge at shared enhancers to coordinate transcriptional activation. In addition, NFATc1 has relatively weak intrinsic DNA-binding affinity and typically requires co-factors such as AP-1 to stabilize promoter occupancy and drive gene expression (12,65–67). The inability of the Wnt SLiM alone to induce CCL2 expression is consistent with a requirement for such cooperative interactions.

Beyond its role as a ligand mimic, TRP120 may further potentiate NFAT-dependent transcription through its HECT E3 ubiquitin ligase activity. We previously demonstrated that TRP120 targets FBW7 during *E. chaffeensis* infection (32). FBW7 regulates AP-1 stability, thereby influencing the formation of NFATc1:AP-1 transcriptional complexes (27). HeLa cells are a tractable model for transfection and were used in this study to investigate the potential of these cellular mechanisms. In the current study, combined activation of NFATc1 via the Wnt SLiM and ectopic expression of TRP120 resulted in enhanced MCP-1 production, supporting a model in which TRP120 integrates extracellular signaling and intracellular regulatory functions. In this model, the TRP120 Wnt SLiM initiates NFATc1 activation and nuclear translocation, while TRP120-mediated modulation of host transcriptional machinery enables full chemokine gene expression. However, elucidating the nuclear interactions and co-regulatory complexes involving TRP120 will be an important focus of future studies.

**Fig 13:**
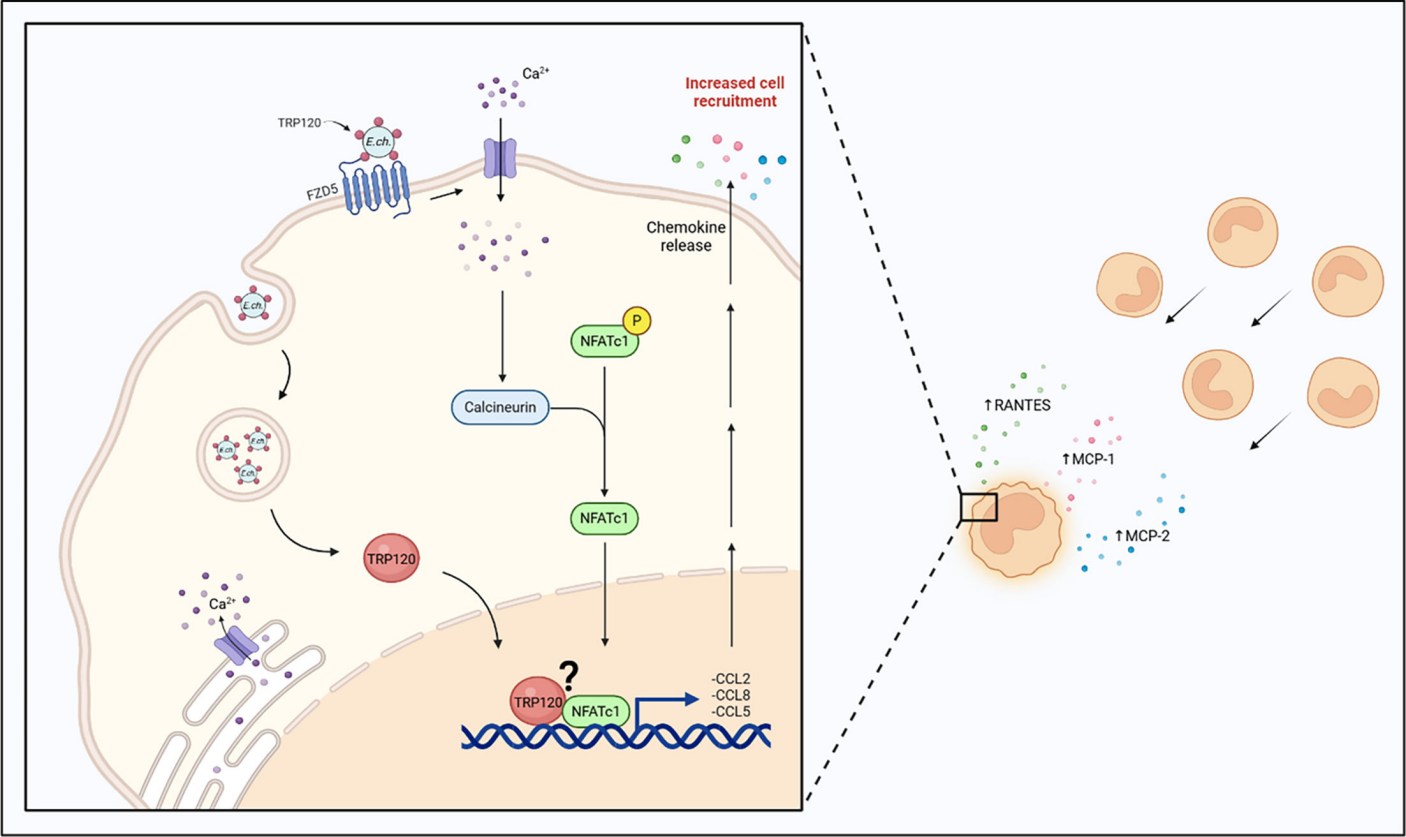
Working model of *E. chaffeensis*-mediated NFATc1 activation, chemokine production, and monocyte chemotaxis. Surface-localized TRP120 Wnt SLiM binds FZD5 and activates non-canonical Wnt/Ca^2+^ signaling, inducing a rapid increase in intracellular calcium. Calcium-dependent calcineurin phosphatase dephosphorylates NFATc1, exposing its nuclear localization sequence that promotes nuclear translocation and activation of downstream target genes. Secreted TRP120 translocates to the nucleus where a previous study demonstrated it binds chemokine genes, likely in complex with NFATc1 or at nearby promoter sites, cooperating to drive chemokine gene expression and subsequent immune cell recruitment.

In summary, we define a mechanism by which a bacterial Wnt ligand mimic activates NFATc1 to drive chemokine production (Fig. 13). This represents the first molecularly defined bacterial-host interaction responsible for chemokine induction. More broadly, these findings highlight the remarkable versatility of TRP120 SLiMs as molecular mimics that integrate multiple host signaling pathways to reprogram cellular responses. This work advances our understanding of *E. chaffeensis* pathogenesis and identifies new potential targets for therapeutic intervention.

## MATERIALS AND METHODS

### Cell culture and *E. chaffeensis* infection

Human acute monocytic leukemia cells (THP-1; ATCC TIB-202) were cultured in RPM1-1640 (30-2001; ATCC) supplemented with 10% fetal bovine serum (FBS; Hyclone SH30910.03) and maintained in a humidified incubator at 37°C with 5% CO_2_ atmosphere. Primary human monocytes were acquired from units of whole blood drawn from deidentified healthy human donors (Gulf Coast Regional Blood Center, Houston, TX) and isolated as previously described (47). *E. chaffeensis* (Arkansas strain) (48) was propagated in THP-1 cells and cell-free ehrlichiae isolated for experiments, as previously described (4). For transfection experiments, human cervical epithelial adenocarcinoma cells (HeLa; CCL-2; ATCC) were propagated in Dulbecco’s modified Eagle’s medium (DMEM; ATCC 30-2002) supplemented with 10% FBS. FZD5 KO cells were commercially generated (Cyagen) using CRISPR/Cas9 technology to introduce a frameshift mutation in *FZD5*. Single colonies were verified by the manufacturer using polymerase chain reaction (PCR) and DNA sequencing. We further confirmed knockout via immunofluorescent microscopy and immunoblot analysis (Fig. S4). FZD5 KO cells were cultured in RPM1-1640 supplemented with 10% FBS.

### Antibodies and peptides

Primary antibodies used for immunofluorescent confocal microscopy and Western immunoblot analysis include monoclonal mouse α-NFATc2 (MA1-025; Invitrogen), monoclonal mouse α-NFATc3 (sc-8405; Santa Cruz Biotechnology), monoclonal mouse α-NFATc4 (sc-271127; Santa Cruz Biotechnology), monoclonal mouse α-NFAT5 (sc-398171; Santa Cruz Biotechnology), monoclonal mouse α-phospho-NFATc1 (MAB5640; R&D Systems), polyclonal rabbit α-FZD5 (PA5-85906; Invitrogen), rabbit α-*E. chaffeensis* disulfide bond formation protein (DSB) (49), rabbit α-TRP120-I1 (50), rabbit α-TRP120-Wnt-SLiM (4), and monoclonal rabbit α-glyceraldehyde 3-phosphate dehydrogenase (GAPDH) (2118L; Cell Signaling Technology). Rabbit α-NFATc1 was commercially generated for use in this investigation (GenScript, Piscataway, NJ) and was derived from previous studies that defined and validated an NFATc1 epitope spanning amino acids 210 to 227 as an effective immunogen for NFATc1 antibody production (51–53). Secondary antibodies used for this study include peroxidase-labeled anti-rabbit IgG (H + L) antibody (5450-0010; KPL), human serum adsorbed and peroxidase-labeled anti-mouse IgG (H + L) antibody (5450-0011; KPL), goat anti-rabbit IgG (H + L) Alexa Fluor Plus 488 (A32731; Invitrogen), goat anti-mouse IgG (H + L) Alexa Fluor Plus 488 (A32723; Invitrogen), and goat anti-rabbit IgG (H + L) Alexa Fluor Plus 594 (A32740; Invitrogen). Soluble peptides for TRP120 Wnt SLiM (QDVASH) and TRP120 Wnt SLiM Mutant (IKDLGAGAGAES; Gly/Ala substitutions in place of the Wnt SLiMs) were commercially synthesized (GenScript). NFATc1 peptide inhibitor, 11R-VIVIT (RRRRRRRRRRRGGGMAGPHPVIVITGPHEE) and scrambled negative control peptide, 11R-VEET (RRRRRRRRRRRGGGMAGPPHIVEETGPHVI) were derived from previous studies (24–26, 35–38, 54), commercially generated, and reconstituted in sterile molecular grade water (GenScript).

### Recombinant proteins

Full-length recombinant TRP120 (rTRP120) and TRP120 Wnt SLiM substitution mutant (rTRP120-SM; Gly/Ala substitutions in place of each Wnt SLiM) were gene synthesized and cloned into pET-32a(+) plasmid constructs (GenScript). One Shot BL21-AI Chemically Competent *E. coli* (C607003; Invitrogen) were transformed with each expression vector by heat shocking in a 42°C water bath for exactly 30 s and placed on ice. Transformed *E. coli* were inoculated in 250 μL of Super Optimal broth with Catabolite repression (SOC) medium (15544034; Invitrogen) and incubated in 37°C for 1 h at 225 rpm. The inoculation mixture was spread onto LB agar plates (113002222; MP Biomedicals) containing 100 μg/mL Ampicillin (A9518; Sigma-Aldrich) and incubated overnight to select for transformed cells. Single colonies were expanded overnight in Terrific Broth (TB; 46-055-CM; Corning), and protein purification was performed as previously described (4). All recombinant protein preparations were dialyzed in cold, sterile-filtered 1× PBS. To ensure minimal endotoxin levels, proteins were processed using a commercially available endotoxin-removal kit in accordance with the manufacturer’s protocol (88274; Thermo Fisher Scientific) and verified with a Limulus Amebocyte Lysate (LAL) test kit (A39552S; Thermo Fisher Scientific). rTRP120 is a thioredoxin-fusion protein; therefore, recombinant Thioredoxin (rThioRx) treatments were included for all experiments as an endotoxin and tag-specific control.

### Immunofluorescent confocal microscopy

THP-1 cells or PHMs harvested for confocal microscopy were washed twice with ice-cold, sterile 1× phosphate-buffered saline (PBS; 500 × *g* for 5 min) and adhered to glass microscopy slides by cytocentrifugation (800 rpm for 5 min). Samples were fixed with 4% paraformaldehyde for 15 min and washed with 1× PBS three times for 5 min. Cells were permeabilized with 0.5% Triton X-100 in 1× PBS for 30 min and washed three times with 1× PBST (0.1% Tween). Cells were then treated with Image-iT FX signal enhancer (Invitrogen) for 30 min, washed three times with 1× PBST, and blocked with BlockAid Blocking Solution (Invitrogen) for 30 min. Primary antibodies were diluted in BlockAid (1:100) and used to incubate samples for 1 h at room temperature (RT). Samples were washed three times with PBST and stained with Alexa Fluor 488/594 secondary antibodies (Invitrogen) diluted in BlockAid solution (1:100) for 30 min at RT, protected from light. Cells were washed three times with PBST and mounted with ProLong Glass Antifade Mountant with NucBlue Stain (Invitrogen). Confocal images were obtained using Zeiss LSM 880 laser microscope and analyzed using Zeiss Zen Blue and FIJI software. Randomized areas per slide were captured at 63× magnification with oil immersion. Mean fluorescent intensities were quantified by designating the nuclear pixels (DAPI, blue channel) as regions of interest (ROI) and superimposing nuclear ROIs to the corresponding pixels for proteins of interest (NFAT, green channel) generating average nuclear pixel intensities for each cell/field.

### Western immunoblot analysis

Whole cell lysates from THP-1 cells were obtained by resuspending harvested cell pellets in RIPA Buffer (R0278; Sigma-Aldrich) containing 1× Halt Protease and Phosphatase Inhibitor Cocktail (Thermo Fisher Scientific) and 2× phenylmethylsulfonyl fluoride (PMSF). Cell suspension was incubated on ice for 30 min with agitation every 10 min. Samples were cleared by centrifugation (14,000 × *g* for 20 min at 4°C) and quantified using bicinchoninic acid assay (BCA). Protein samples were mixed with 4× NuPAGE LDS Sample Buffer (Invitrogen) and 1× NuPage Sample Reducing Agent (Invitrogen) and boiled for 5 min at 95°C and placed on ice. Equal amounts (25 μg/well) were loaded and separated by sodium dodecyl sulfate-polyacrylamide gel electrophoresis (SDS-PAGE) and transferred onto nitrocellulose membrane. Membranes were blocked with EveryBlot Blocking Buffer (Bio-Rad) and probed with primary (1:1,000; overnight at 4°C) and secondary antibodies (1:10,000; 1 h) diluted in EveryBlot. Membranes were washed three times with 1× Tris-buffered saline with 0.1% Tween 20 (TBST) and incubated with WesternBright Sirius Chemiluminescent Detection Kit (K-12045-D50; Advansta). Image acquisition was performed with the ChemiDoc MP Imaging System (Bio-Rad) followed by densitometry quantification with VisionWorks analysis software.

### TRP120 Wnt SLiM antibody neutralization assay

Cell-free *E. chaffeensis* was premixed with 1.5 μg/mL of rabbit α-TRP120-Wnt-SLiM antibody or rabbit preimmune serum (negative control). *E. chaffeensis*/serum mixtures were then incubated for 2 h in 4°C and then added to THP-1 cells for 3 h. Rabbit α-TRP120-Wnt-SLiM was commercially generated against a TRP120 epitope spanning aa 84 to 104 that contains the Wnt SLiM mimic (4).

### RNA extraction and cDNA synthesis

Total RNA from uninfected, *E. chaffeensis-*infected, rTRP120-treated, and rThioRx-treated THP-1 cells was isolated using RNeasy Mini Kit (Qiagen), and DNA digestion was performed using RNase-Free DNase Set (Qiagen) according to the manufacturer’s protocols. RNA concentration and purity were measured with the NanoDrop 100 spectrophotometer (Thermo Fisher Scientific). Total RNA (0.5 μg) was used to synthesize cDNA with iScript cDNA Synthesis Kit (Bio-Rad) as per the manufacturer’s protocol.

### Human cytokine and chemokine PCR array

The RT^2^ Profiler Human Cytokine & Chemokine PCR Array (PAHS-150Z; Qiagen) targeted the expression of 84 genes. PCR arrays were performed following the manufacturer’s handbook with the addition of Brilliant II SYBR Green QPCR Master Mix (Agilent). Real-time qPCR was conducted on the QuantStudio 6 Flex real-time PCR system (Thermo Fisher Scientific), with run conditions and assay analysis performed as previously described (4).

### Dot blot membrane array

Conditioned media from uninfected and *E. chaffeensis*-infected THP-1 cells (pre-treated with 11R-VIVIT or 11R-VEET) were harvested and incubated with membranes from the Human Cytokine Array Kit (RayBiotech; AAH-CYT-5-4), prepared with 80 capture antibody targets. Secreted cytokines and chemokines were screened according to the manufacturer’s protocol and imaged with the ChemiDoc MP Imaging System (Bio-Rad) followed by densitometry quantification with VisionWorks analysis software.

### Enzyme-linked immunosorbent assay

Conditioned medium was harvested from uninfected, *E. chaffeensis*-infected THP-1 cells (pre-treated with 11R-VIVIT or 11R-VEET) and FZD5 KO cells. ELISA assays were performed for MCP-1/CCL2 (DCP00; R&D Systems), MCP-2/CCL8 (DRN00B; R&D Systems), and RANTES/CCL5 (NBP2-68059; R&D Systems) according to the manufacturer’s protocols. Results were measured using basic endpoint analysis on the VersaMax microplate reader (Molecular Devices) and analyzed with SoftmaxPro software (Molecular Devices).

### Transwell migration assay

Serum-starved THP-1 cells were seeded (5 × 10^5^ cells/mL; 200 μL) in cell culture inserts equipped with 5 μM porous membranes (9325012; Fisher) and placed in a 24-well plate. Bottom chambers were filled with conditioned media (900 μL) from uninfected, *E. chaffeensis*-infected THP-1 cells (pre-treated with 11R-VIVIT or 11R-VEET) and FZD5 KO cells harvested 48 hpi. Plates were incubated at 37°C with 5% CO_2_ for 2 h, inserts were removed, and live cell imaging of the bottom chamber was performed using the ImageXpress Pico (Molecular Devices) at 4× magnification and enumerated by cells/field.

### Transfection

HeLa cells (2 × 10^5^ cells/mL) were seeded in a 12-well plate 24 h prior to transfection. Lipofectamine 3000 transfection kit (L3000015; Invitrogen) was used according to manufacturer’s protocol. Briefly, Lipofectamine was diluted in Opti-MEM and combined with DNA plasmid mixture (pAc-GFP or pcDNA3.1-6xHis) containing Opti-MEM, TRP120 or empty vector, and P3000 reagent and incubated at RT for 15 min. Lipofectamine/DNA plasmid mixtures were added dropwise to HeLa cells. Medium was replaced post transfection (4 h) and incubated at 37°C with 5% CO_2_ for 48 h.

### Statistical analysis

All data were collected from at least three independent biological and technical replicates and represented as averages ± standard deviation. Statistical significance of pairwise comparisons is indicated in each figure and was assessed using either two-tailed Student’s *t*-test or one-way analysis of variance (ANOVA) with Dunnett’s *post hoc* test to compare all treatment groups to control group or Bonferroni’s *post hoc* test for multiple comparisons across treatment groups. Analyses were conducted on GraphPad Prism 10 software with *P* < 0.05 considered statistically significant.

## SUPPLEMENTAL MATERIAL

Supplemental figures msphere.00081-26-s0001.docx Figures S1 to S5.

Table S1 msphere.00081-26-s0002.xlsx Complete gene list and RT qPCR expression analysis for human cytokine and chemokine genes.

Table S2 msphere.00081-26-s0003.xlsx Analyte map of multiplex dot blot chemokine array used to screen expression levels of 80 chemokines during *E. chaffeensis* infection.

ASM does not own the copyrights to Supplemental Material that may be linked to, or accessed through, an article. The authors have granted ASM a non-exclusive, world-wide license to publish the Supplemental Material files. Please contact the corresponding author directly for reuse.
