# Supplementary material for: TRP120-dependent activation of noncanonical Wnt/NFAT signaling drives monocyte chemokine production in Ehrlichia chaffeensis infection
Source: mSphere. 2026 Jun 22;11(7):e00081-26. doi: 10.1128/msphere.00081-26 (PMC13410762; doi:10.1128/msphere.00081-26)
Supplement: Supplemental figures — Figures S1 to S5. [file msphere.00081-26-s0001.docx]

**FIG S1. NFATc1 activation kinetics throughout *E. chaffeensis* infection time course**

Immunofluorescent confocal microscopy of *E. chaffeensis*-infected (MOI 100) THP-1 cells harvested at indicated timepoints and stained with NFATc1 to detect nuclear localization. Subsequent experiments were conducted between 0 – 6 hpi to isolate cellular phenotypes with peak NFATc1 activation.

A

B

**FIG S2. Cell viability analysis for optimal inhibitor concentration.**

THP-1 cells were treated with the indicated concentrations of (A) 11R-VIVIT and (B) 11R-VEET and harvested 48 hpt to determine cell viability. Annexin V flow cytometry analysis was used to identify the percentage of live, early/late apoptotic, and dead cells in response to inhibitor.

**FIG S3. SDS-PAGE of purified recombinant TRP120 and substitution mutant**.

Lane 1, molecular weight marker; 2) Full length TRP120 (TRP120-FL); 3) full length TRP120 mutant (TRP120-SM); 4) Thioredoxin (negative control) stained with Coomassie blue.

**FIG S4. Confirmation of FZD5 knockout cell line**.

Immunofluorescent microscopy and western blot analysis of commercially generated FZD5 gene knockouts in THP-1 cells. All experiments were conducted using FZD5 KO 1G1.

B

A

**FIG S5. Verification of HeLa transfection efficiency.**

Western immunoblot analysis of whole cell lysates probed with α-TRP120. Transfection efficiency was further confirmed using (A) α-GFP or (B) α-6xHis antibody to detect wild-type and mutant TRP120 expression.
